# Mastitis as a risk factor for breast cancer: a systematic review and meta-analysis

**DOI:** 10.1007/s12094-026-04303-x

**Published:** 2026-04-12

**Authors:** Aryane Cristina de Figueiredo Azevedo, Raissa Martins da Silva, Felipe Alves de Paiva, Luis Henrique Rios Moreira Rego, Francisco Cezar Aquino de Moraes

**Affiliations:** 1 Federal University of Alfenas, Alfenas, Minas Gerais 37130000 Brazil; 2 Federal University of Santa Catarina, Florianópolis, Santa Catarina 88040-900 Brazil; 3 Federal University of Amazonas, Manaus, Amazonas 69077-000 Brazil; 4 State University of Piaui, Teresina, Piaui 64001280 Brazil; 5 University of São Paulo, Sao Paulo, 05508-000 Brazil

**Keywords:** Breast cancer, Mastitis, Risk factor, Cancer epidemiology, Meta-analysis

## Abstract

**Background:**

Breast cancer (BC) is the most common cancer among women worldwide, accounting for more than 2.3 million new cases annually. Although chronic inflammation is a well-established risk factor for cancer, the association between mastitis and BC has not yet been clearly established. Therefore, this study aims to investigate mastitis as a potential risk factor for BC.

**Methods:**

We performed a random-effects meta-analysis to estimate the odds ratio (OR) and hazard ratio (HR). P values lower than 0.05 were considered statistically significant, with 95% confidence intervals (CI). Statistical analyses were conducted using R Studio software version 4.4.2, considering P < 0.05 as statistically significant.

**Results:**

Our meta-analysis included 54,216 patients, of whom 18,753 (34.6%) had mastitis, while 35,463 (65.4%) comprised the control group. Our results support that the risk of BC is higher among patients with a history of mastitis (OR: 2.12; 95% CI: 1.44–3.11; p = 0.0423). Similarly, longitudinal analysis based on total follow-up also confirmed an increased risk of BC in the mastitis group (HR: 2.0057; 95% CI: 1.1739–3.4269; p = 0.011).

**Conclusion:**

This meta-analysis highlights a possible association between the presence of mastitis and BC, substantially contributing to the advancement of understanding regarding risk factors for this cancer. Prospective studies using robust detection methods and paired tissue analyses are needed to confirm these findings and clarify the role of mastitis in breast tumorigenesis.

**Graphic abstract:**

**Figure:**
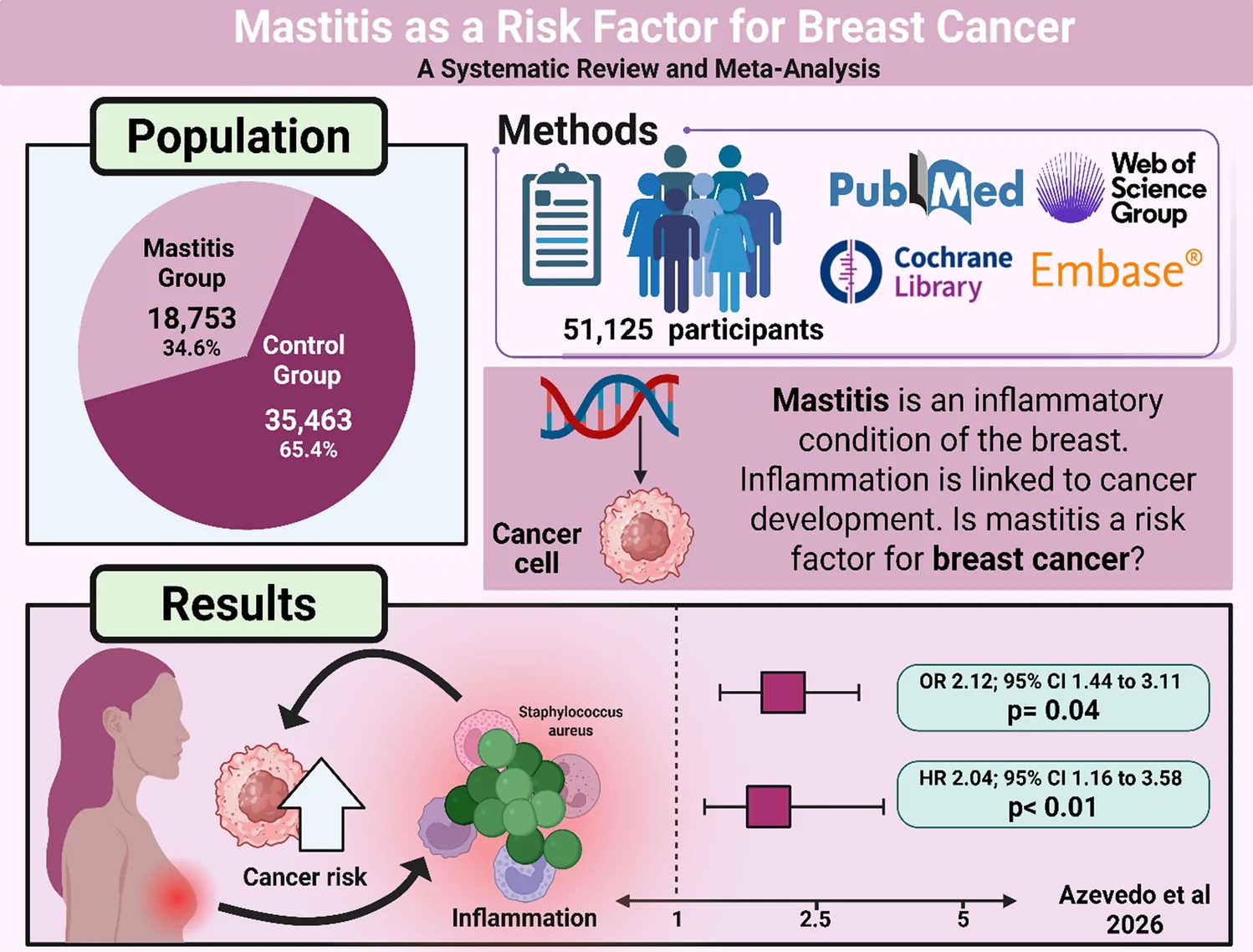

**Supplementary Information:**

The online version contains supplementary material available at https://doi.org/10.1007/s12094-026-04303-x.

## Introduction

BC is the most prevalent malignant neoplasm in women worldwide. According to data from the International Agency for Research on Cancer (IARC) in 2022, the incidence of the neoplasm was approximately 2.3 million new cases, with an estimated 666,000 deaths globally [1, 2]. Several risk factors are known to increase the likelihood of tumor occurrence, such as early menarche, late menopause, hormone replacement therapy, family history, genetic mutations, low parity, advanced age, and lifestyle changes, including alcohol consumption, among others [3–5].

According to the World Health Organization (WHO), mastitis is an inflammatory condition that may or may not be associated with infection. In clinical practice, it is most commonly observed during lactation, which is why it is referred to as lactational or puerperal mastitis [6]. On the other hand, non lactational mastitis is commonly classified into specific subtypes, with periductal mastitis being the subtype most frequently associated with smoking and, in some cases, bacterial infections [6]. Several studies have shown that inflammation, although a common response of the human body, acts as a pro-tumor agent. Through the release of biochemical and hematological markers, it influences the occurrence of malignant tumors, promoting the proliferation and survival of neoplastic cells in the tumorigenesis process, and also affecting prognosis and mortality [7]. Tumors such as endometrial, ovarian, and colorectal cancers have already been linked to chronic inflammatory processes as a risk factor for the development of neoplasia [8, 9].

Although mastitis is a common disease, there is still no clear evidence in the literature as to whether it can be considered a potential risk factor for the development of BC. Thus, this meta-analysis aims to review articles that assess this aspect and determine whether there is a new risk factor for the development of this neoplasm, which impacts women's health.

## Methods

### Protocol and registration

This meta-analysis was conducted in adherence to the Preferred Reporting Items for Systematic Reviews and Meta-Analyses (PRISMA) [10] guidelines and aligned with the recommendations outlined by the Cochrane Collaboration [11]. The review is registered with the Prospective International Registry of Systematic Reviews (PROSPERO) under registration number CRD42025648010.

### Eligibility criteria

Studies were included if they met the following eligibility criteria: case–control (CC) or cohort studies (CS) involving female patients aged 18 years or older diagnosed with lactational or non lactational mastitis (NLM) at any time in their life. Lactational mastitis refers to cases of women affected by the infection during the breastfeeding period, while non-lactational mastitis is the inflammatory/infectious process occurring outside the breastfeeding period, affecting women who have never breastfed or who are no longer lactating at the time. Exclusion criteria included studies with overlapping populations, those without a control group, case reports, reviews, opinion pieces, technical reports, guidelines, and animal studies. Additionally, only articles published in English after the year 2000 were considered.

### Search strategy

We performed a systematic search of published studies using PubMed, Web of Science, Cochrane Central, and Scopus through 15 December 2024. The search strategy was independently executed by two authors (A.C.F.A. and R.M.S.). To identify additional studies, we also reviewed the references of included articles, abstracts, and systematically analyzed relevant literature reviews. The following terms were used: “breast cancer”, “mastitis”, “risk”, “association”, “women”, “lactant women”, “non lactating women.” For each database, search terms were tailored to its specific syntax rules, incorporating both Medical Subject Headings (MeSH) terms and free-text keywords, combined using Boolean operators (OR, AND). The detailed search strategy used is detailed in the supplementary material (Table S4).

Studies identified through database searches and article references were imported into Rayyan's reference management software [12]. Duplicate entries were removed using a combination of automated and manual screening. Titles and abstracts of the retrieved articles were independently reviewed by two authors (A.C.F.A. and R.M.S.), who also independently extracted data based on predefined search criteria and quality assessment protocols. Any discrepancies between the reviewers were resolved by consultation with a third reviewer, who made the final decision on inclusion.

### Data extraction and risk of bias assessment

To synthesize the key findings, two authors (F.A.P. and B.D.D.) independently compiled the data extracted from the included studies. This included details such as authorship and publication year, study design, patient sample characteristics (total number of women studied, age, duration of study). We prioritized adjusted values when available.

The quality assessment of observational studies was performed using the Newcastle–Ottawa Scale (NOS), which scores studies on a 0 to 9 scale according to selection, comparability, and exposure criteria [13]. High quality is considered when studies considered 8 or 9. Two authors (F.A.P and R.M.S) independently conducted the risk of bias assessment and disagreements were resolved by a third author. We also used the leave-one-out method and Baujat graphic to identify studies with a disproportionate influence on the overall effect size or heterogeneity [14]. Each trial was evaluated in different domains, namely: selection of exposed cohorts, external controls, exposures and outcomes of interest; comparability of main and additional factors; outcome assessment, follow-up time, and adequacy of follow-up time.

### Statistical analysis

Treatment effects for binary endpoints, such as diagnosis of BC, were compared using pooled odds ratio (OR) with 95% confidence intervals. For hazard outcomes, Hazard ratios (HRs) with corresponding 95% confidence intervals (CIs) were computed with inverse variance random-effects model. Heterogeneity was assessed using the I^2^ and Tau^2^ statistics. We used a fixed-effect model for endpoints with I^2^ < 25% and DerSimonian and Laird random-effect models for pooled outcomes with high heterogeneity. Statistical analyses were performed using R statistical software, version 4.4.2 (R Foundation for Statistical Computing).

## Results

**Fig. 1:**
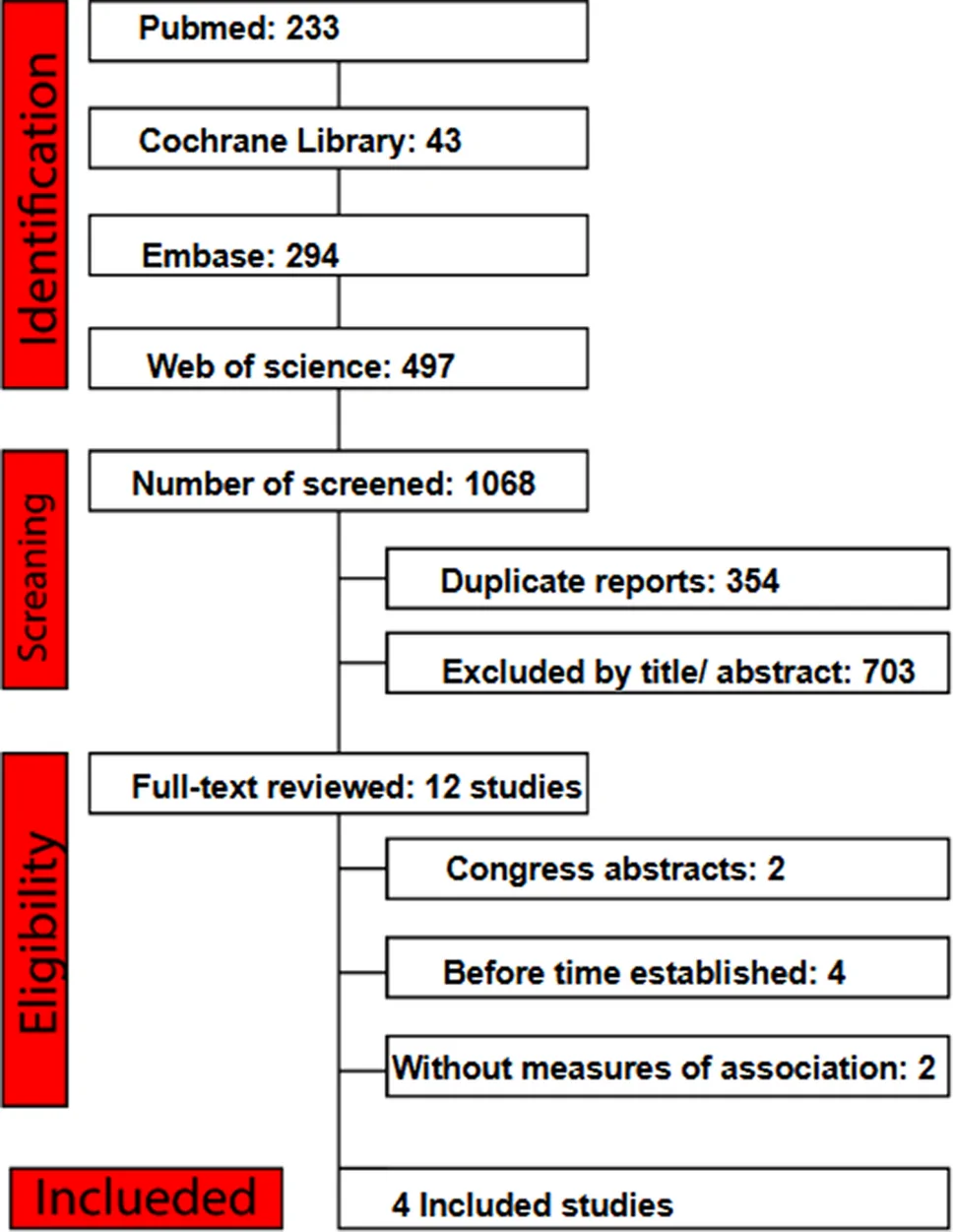
PRISMA flow diagram

As illustrated in the PRISMA flow diagram (Fig. 1), the systematic search identified 1068 references. After removing duplicate records and screening titles and abstracts, 12 full-text articles were assessed for eligibility based on predefined inclusion and exclusion criteria. Four observational studies met the eligibility criteria and were included in the final analysis [15–18].

The individual characteristics of the studies are summarized in Table 1. A Total of 54,216 women were included, 18,753 in the Mastitis group (MG) and 35,463 in the Control group (CG). We included three Retrospective cohorts (RC) and one CC. The mean time of duration was eight years. The predefined inclusion and exclusion criteria of each study is detailed in the supplementary material Table S3.

**Table 1:** Characteristics of included studies

Study (year)	Design	Country	Total number of women studied	Women with mastitis/women without mastitis	Age	Comorbidity	Duration of study
Krishnan, 2024 [16]	Retrospective cohort study	Germany	29.784	14.892/14.892	≥ 18 years old	Obesity	2005–2021
Bothou, 2022 [15]	Case control study	USA	343	36/307	NA	NA	2017–2020
Chen, 2020 [17]	Retrospective cohort study	Taiwan	8.634	734/7.900	≥ 40 years old	Hypertension, hyperlipidemia, diabetes, hyperthyroidism, hypothyroidism, autoimmune diseases	2010–2012
Chang, 2019 [18]	Retrospective cohort study	Taiwan	15.455	3.091/12.364	≥ 20 years old	Schizophrenia, hypertension, chronic obstructive pulomnary disease, thyroid disease, diabetes, hyperlipidemia, obesity	2000–2011

*NA* not available

### Incidence of breast cancer

**Fig. 2:**
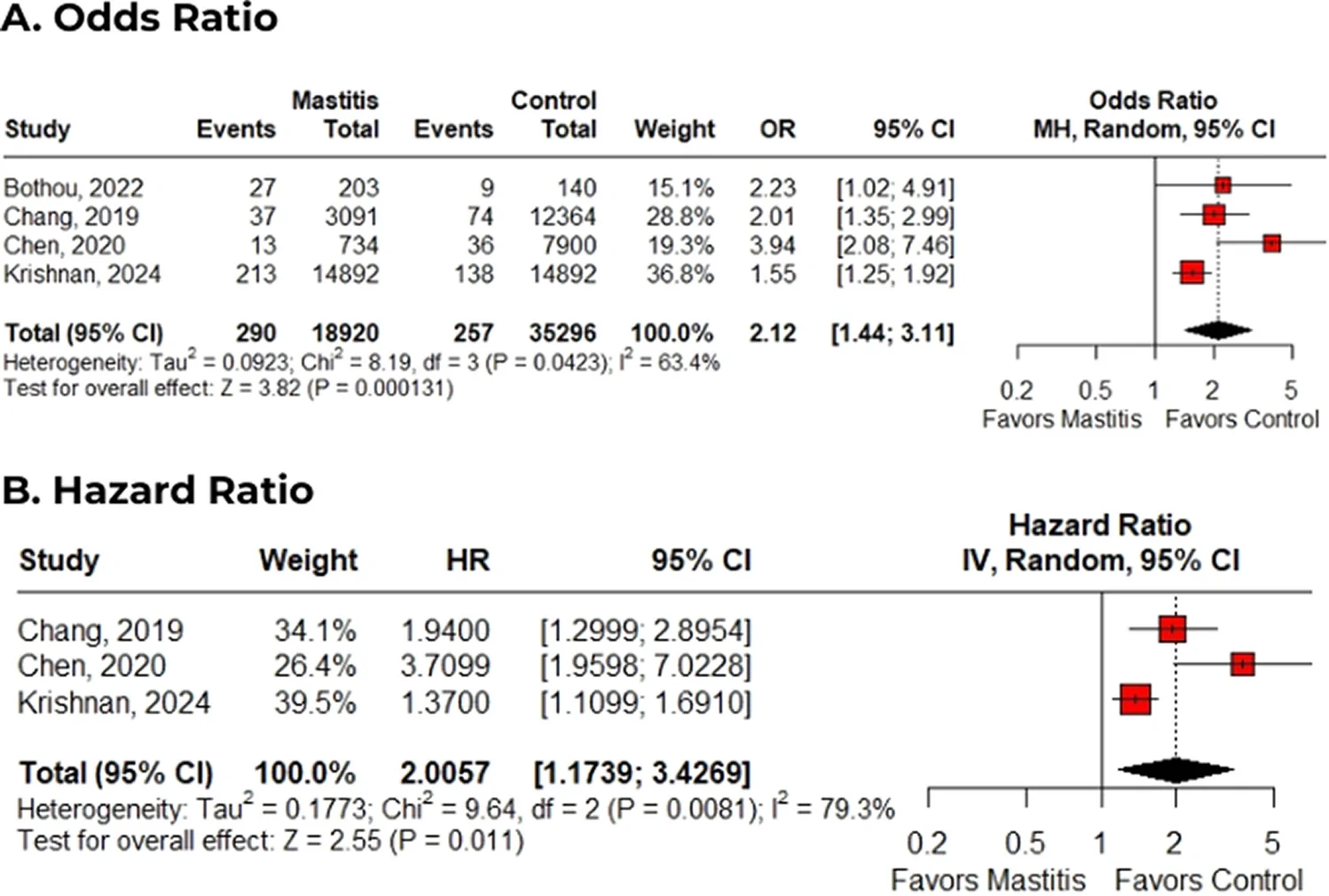
Forest plot for incidence of BC odds ratio (**A**) and hazard ratio (**B**)

Four observational studies [17–20] involving 54,216 patients assessed the odds ratio for BC in individuals in MG compared to those in CG. The pooled analysis revealed a statistically significant association with an OR 2.12; 95% CI 1.44–3.11; (p = 0.0423); I^2^ = 63.4%; (Fig. 2A), indicating a possible higher risk of BC among individuals with a history of mastitis.

Three retrospective cohort studies [18–20], involving 53,873 women, analyzed the hazard ratio (HR) and found that the risk of developing BC over time was higher in MG compared to those in CG. The pooled analysis revealed a statistically significant association between BC and Mastitis, with a HR 2.0057; 95% CI 1.1739–3.4269; (p = 0.011). Heterogeneity is considered substantial (I^2^ = 80.7%) (Fig. 2B).

#### 3.2.1 Quality assessment and Risk of Bias.

**Fig. 3:**
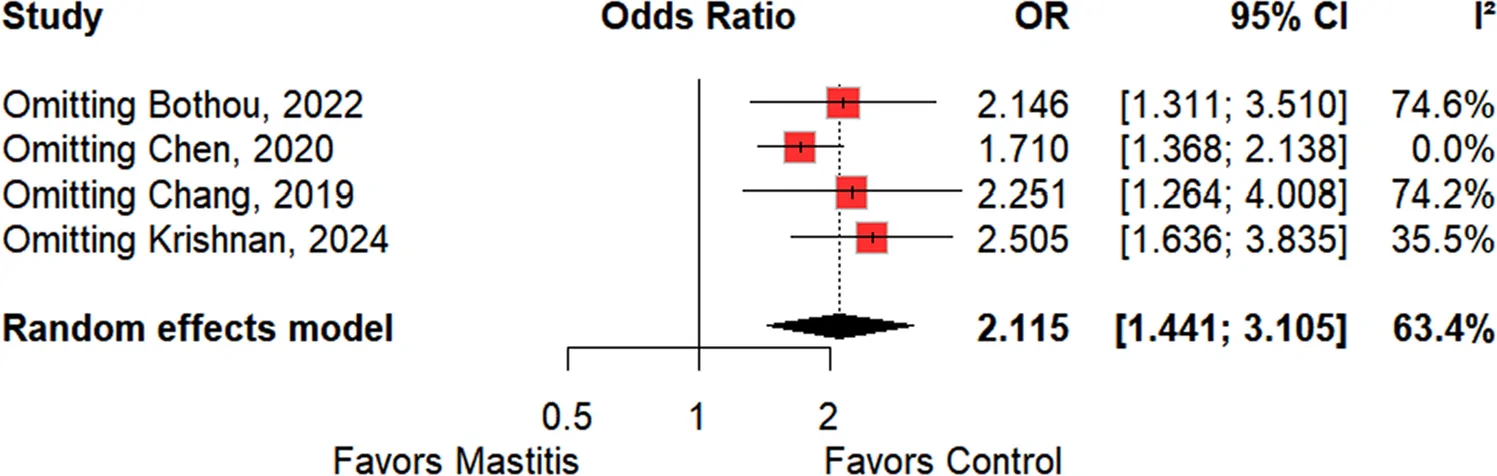
The leave-one-out for incidence of BC

Leave-one-out analysis was conducted for the Incidence of BC outcome, due to an I^2^ > 25%, indicating heterogeneity. The exclusion of individual studies did not significantly alter the pooled estimates, with the pooled OR ranging from 1.71 to 2.51, confirming the robustness of the findings. Analyzing the graphic, the omission of Chen et al. reduces heterogeneity to zero, while the omission of Krishnan et al. considerably reduces it (Fig. 3).

**Fig. 4:**
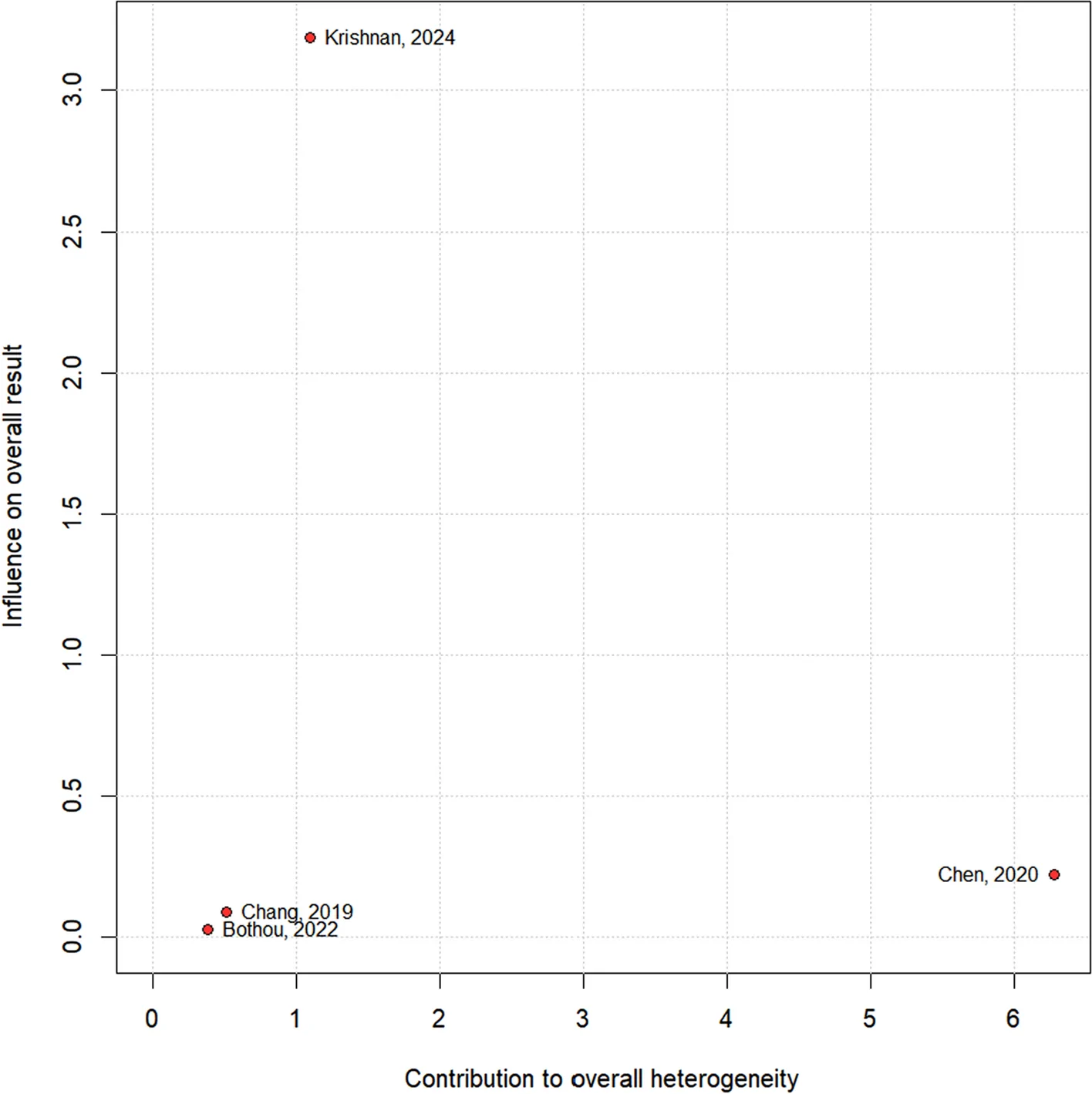
Baujat analysis for incidence of BC

The Baujat analysis identified that Chen et al., 2020 [17] was found to be the major source of heterogeneity, while Krishman et al., 2024 significantly influenced the overall results for incidence of BC (Fig. 4).

According to the Newcastle–Ottawa Scale (NOS), two studies Krishnan et al., 2024 and Chen et al., 2020 were classified as high quality. Chang et. al 2019 and Bothou et.al, 2022, were rated as low quality (Table 2). Chang et. al lost points in the comparability criteria between populations due to the lack of a secondary outcome, and the lack of an adequate flow-up time. Bothou et. al being a case–control study, it conducted a retrospective analysis starting from the outcome of interest. It also loses points for not having a representative sample of the population, for not providing data on additional outcomes analyzed, and for not presenting an adequate follow-up period.

**Table 2:** Methodological quality summary using Newcastle Ottawa Scale

Meta-data			Newcastle–Ottawa Scale			
Author	Publication date	Origin	Selection	Comparability	Outcome	Quality
Krishnan	2024	Germany	☆☆☆☆	☆☆	☆☆☆	8
Chen	2020	Taiwan	☆☆☆	☆☆	☆☆	8
Chang	2019	Taiwan	☆☆☆☆	☆	☆☆	6
Bothou	2019	USA	☆☆	0	☆☆	4

Methodological quality summary using Newcastle Ottawa Scale. Maximum quality score = 9; 0–7 points were considered lower quality, and 8–9 points were considered as higher quality

## Discussion

In this systematic review our objective was to verify whether women who experienced mastitis at any point in their lives had a higher risk of developing BC. The studies included analysed women diagnosed with LM and NLM. According to our results, there may be a association between the two factors, positioning mastitis as a potential risk factor for the development of BC (p = 0.0423).

Since the beginning of the twentieth century, there have been works indicating a possible correlation between mastitis and BC [19]. More recently, Nolan et al. [20] published a literature review on the subject, presenting hypotheses and possible pathophysiological mechanisms on how mastitis associates wih BC. Some case reports describe patients with chronic mastitis who concurrently developed BC [21]. In studies with more representative populations, Lamble et al. (2009) found that women affected by mastitis had an increased incidence of BC compared to women who did not have the infection [22]. Peters et al. followed women with non-lactational mastitis over one year and compared their incidence of BC to that of the general population. Conversely, in our study, it was found that women affected by NLM had a higher incidence of the neoplasm [23].

Recent studies have indicated a strong association between neoplasms and certain infectious agents and inflammation. In 2016, Zhu et al. [24] published a meta-analysis showing a significant correlation between *Chlamydia* infection and cervical cancer, considering it an independent risk factor for this type of tumor (OR = 1.76, 95% CI: 1.03–3.01, P = 0.04). Similarly, Ford et al. [25] published a study in 2020 demonstrating that the eradication of *H. pylori* significantly reduces the incidence of gastric cancer in healthy individuals (RR = 0.54; 95% CI 0.40–0.72, NNT = 72). In the field of inflammation, Michels et al. [26] showed that the presence of inflammatory markers in the body increases the incidence of BC (OR = 1.23 [1.05–1.43]; HR = 1.14 [1.01–1.28]). Guo et al. [27] also confirmed this correlation and demonstrated that it is stronger in Asian populations (OR = 1.57, 95% CI: 1.25–1.96) compared to the American population (OR = 1.08, 95% CI: 1.01–1.16) and European population (OR = 1.12, 95% CI: 1.02–1.23). Correlating the above data with the hypothesis of this work, our findings suggest that mastitis may be associated with increased breast cancer risk. However, causality cannot be inferred from observational data (OR 2.12; 95% CI 1.44–3.11).

Several studies support that inflammation is one of the key contributing factors of neoplasms. Obesity, a well-established risk factor for BC, exerts its effect primarily through the inflammation caused by excess adipose tissue in the body, resulting in the release of inflammatory cytokines such as TNF-α and IL-6 [28]. These same cytokines are released in the pathophysiological process of mastitis [29]. TNF-α acts through positive feedback for NF-kB, which promotes tumor cell proliferation, as well as stimulating estrogen production, a risk factor that is associated with increased risk of BC [30]. IL-6, which is the main cytokine elevated in mastitis and has been considered a biomarker of the severity of this pathology, also plays a role in the process of oncogenesis and releases factors for tumor maintenance and growth [31]. However, these mechanistic considerations are hypothesis-generating and should be interpreted as speculative, since the available epidemiologic evidence remains limited and not yet sufficient to support causal inference.

If mastitis contributes to breast carcinogenesis, the most biologically plausible mechanism underlying this association appears to be persistent inflammation triggered by an infectious agent. Accordingly, sustained inflammatory cellular signaling may be associated with cellular senescence and proliferation, particularly during repeated cycles of tissue injury and repair, which can increase oxidative stress and lead to consequent cellular DNA damage. In addition, this inflammatory process may induce stromal remodeling and angiogenesis that could contribute to neoplastic development. These processes may be mediated by cytokines and pro inflammatory pathways such as NF κB. Although this framework could partially justify an increased cancer risk, this hypothesis is purely speculative, as the current epidemiologic evidence does not yet support a validated mechanistic basis.

Another factor that may increase BC incidence in patients affected by mastitis is the alteration of breast microbiota. Several studies have suggested that different types of bacteria can influence cancerous tumors, as in the case of H. pylori in gastric cancer [32, 33] and chlamydia in cervical cancer [34]. A study conducted by Jin et al. [35] in 2023 showed a significant increase in streptococcus in human milk samples from women affected by mastitis. Jimenez et al. conducted a study on human milk from women with mastitis and healthy women, and a considerable reduction in bacterial diversity was observed in the mastitis group [36]. This decrease in diversity was also noted in patients with BC in a study conducted by Heineken et al. [37]. In contrast, Rad et al. [38] published a meta-analysis in 2025 analyzing the differences between the microbiota of normal breast tissues, those affected by mastitis, and those affected by BC. Regarding mastitis and BC, it was found that tissues affected by mastitis exhibited a greater diversity of microbiota when compared to tissues affected by BC, as well as a difference in the overall microbiota composition between the groups. However, according to analyses, this difference may be more due to the heterogeneity within each group rather than being truly different between the two compared groups. Similarly, a meta-analysis conducted by Moraes et al. [39]. analyzing 5,133 BC tissues revealed that infection with Epstein-Barr virus (EBV) may be a risk factor for BC, with its presence being significantly associated with an increased risk of developing the disease (RR: 3.35; P < 0.001). Although there are some divergences, it is observed that the alteration in the microbiota due to mastitis may be related to the increased risk of BC, even though the pathophysiological mechanisms are not yet fully understood. This reinforces the importance of future studies to verify whether there is indeed a causality and the mechanisms involved.

Our systematic review has some limitations. First, the modest number of studies included, with only one outcome analyzed, with a high heterogeneity, reflecting a significant discrepancy in the characteristics of the included patients. Second, there was a lack of baseline characteristics of the population in some included studies which is expected, given that the included studies are observational, and, in most cases, the authors do not pay as much attention to these population characteristics as in interventional studies. This fact creates an important limitation to our study, as without information on the population's characteristics, such as obesity, parity, smoking, among others, it is not possible to conduct statistical analyses that reveal other factors that may influence our results. Third, some papers had a greater influence on the resultant analyses, mainly represented by Krishnan et al. which covered a larger sample size when compared to the other included studies. However, when we evaluate the weight of studies using the leave one out method, we see that there are no significant changes in the main outcome even with its omission. Fourth, a significant asymmetry in the results was observed, which may be due to discrepancies in the analysis methods and significant differences in the number of participants included. Although there was no significant difference in the results, as verified by the leave-one-out method, this ultimately increases the risk of bias. Fifth we did not find prospective studies about this subject, all included studies were retrospective, with these findings underscore the importance of conducting further research, leading to the initiation of prospective studies. Sixth, the risk of bias evidence that some studies presented low quality. Despite these points, our general findings suggest consonant results in the resultant analyses, supporting the reliability of our conclusion, in addition to the leave-one-out analysis demonstrating a similar trend.

A critical point that should be considered when interpreting our meta-analysis is surveillance or detection bias. Indeed, women diagnosed with mastitis tend to undergo a greater number of investigations for breast evaluation, including imaging examinations, clinical follow up, and even invasive procedures such as breast tissue biopsy. This could lead to a potential increase in the diagnosis of asymptomatic breast cancer in the mastitis group, particularly in the short term after the observed inflammatory process, which may artificially inflate the absolute frequency of cancer detected. Therefore, we acknowledge that breast cancer cases in the mastitis group could reflect higher detection rates driven by increased use of diagnostic breast procedures rather than a direct causal relationship, since most included studies did not adequately adjust for health care utilization, screening frequency, risk groups, or the time window between mastitis and breast cancer diagnosis. Accordingly, our findings should not only be interpreted with caution but also validated in future prospective cohorts that systematically adjust for diagnostic surveillance, risk factors, and follow up to reduce the risk of bias.

## Conclusion

This review suggests a potential association between mastitis and an increased risk of breast cancer. More studies must be conducted on the subject to better investigate the correlation between comorbidities and to create strategies for prevention, treatment, and to include new screening strategies in the affected population. Further research should aim to better characterize the studied populations and risk factors providing more data on the subject and reiterate the hypothesis of this study.

## CRediT authorship contribution statement

**Aryane Cristina de Figueiredo Azevedo**: Conceptualization, Methodology, Investigation, Data curation, Writing – original draft, Visualization, Figure preparation. **Raissa Martins da Silva**: Data collection, Formal analysis, Writing – original draft, Figure preparation. **Felipe Alves de Paiva**: Data collection, Analysis, Visualization, Writing – original draft, Table preparation. **Luis Henrique Rios Moreira Rego**: Writing – review & editing, Writing – original draft, Validation. **Francisco Cezar Aquino de Moraes**: Supervision, Methodology, Project administration, Conceptualization, Writing – review & editing, Validation. All authors contributed to the study conception and design, commented on previous versions of the manuscript, and approved the final version.

## Supplementary Information

Below is the link to the electronic supplementary material.

Supplementary file1 Supplementary Material: The supplementary material provides detailed information on the search strategy, subgroup analyses (eFigures), sensitivity analyses (leave-one-out), and the quality assessment of the included studies. Additional data may be made available upon request to the corresponding author. (DOCX 8865 KB)
