# Supplementary material for: Mastitis as a risk factor for breast cancer: a systematic review and meta-analysis
Source: Clin Transl Oncol. 2026 Apr 12;28(10):4657–65. doi: 10.1007/s12094-026-04303-x (PMC13601124; doi:10.1007/s12094-026-04303-x)
Supplement: Supplementary file 1 [file 12094_2026_4303_MOESM1_ESM.docx]

**Supplementary Material**

**Supplementary Table 1.** PRISMA 2020 Checklist.

**Supplementary Table 2.** PRISMA 2020 for Abstract Checklist.

**Supplementary Table 3.** Inclusion and exclusion criteria of included studies.

**Supplementary Table 4.** Search Strategies.

**Supplementary Table 1.** PRISMA 2020 Checklist.

| Section and Topic | Item # | Checklist item | | Location where item is reported |
| --- | --- | --- | --- | --- |
|  |  |  | |  |
| TITLE | | | |  |
| Title | 1 | Identify the report as a systematic review. | | Page  1 |
| ABSTRACT | | | |  |
| Abstract | 2 | See the PRISMA 2020 for Abstracts checklist. | | Supplementary Materials: |
| INTRODUCTION | | | |  |
| Rationale | 3 | Describe the rationale for the review in the context of existing knowledge. | | Page 3 |
| Objectives | 4 | Provide an explicit statement of the objective(s) or question(s) the review addresses. | | Page 3-4 |
| METHODS | | | |  |
| Eligibility criteria | 5 | Specify the inclusion and exclusion criteria for the review and how studies were grouped for the syntheses. | | Pages 5 |
| Information sources | 6 | Specify all databases, registers, websites, organisations, reference lists and other sources searched or consulted to identify studies. Specify the date when each source was last searched or consulted. | | Page 5-6 |
| Search strategy | 7 | Present the full search strategies for all databases, registers and websites, including any filters and limits used. | | Supplementary Materials: Table S3 |
| Selection process | 8 | Specify the methods used to decide whether a study met the inclusion criteria of the review, including how many reviewers screened each record and each report retrieved, whether they worked independently, and if applicable, details of automation tools used in the process. | | Page 5-6 |
| Data collection process | 9 | Specify the methods used to collect data from reports, including how many reviewers collected data from each report, whether they worked independently, any processes for obtaining or confirming data from study investigators, and if applicable, details of automation tools used in the process. | | Page 5-6 |
| Data items | 10a | List and define all outcomes for which data were sought. Specify whether all results that were compatible with each outcome domain in each study were sought (e.g. for all measures, time points, analyses), and if not, the methods used to decide which results to collect. | | Pages  6 |
|  | 10b | List and define all other variables for which data were sought (e.g. participant and intervention characteristics, funding sources). Describe any assumptions made about any missing or unclear information. | | Page 6 |
| Study risk of bias assessment | 11 | Specify the methods used to assess risk of bias in the included studies, including details of the tool(s) used, how many reviewers assessed each study and whether they worked independently, and if applicable, details of automation tools used in the process. | | Page 6 |
| Effect measures | 12 | Specify for each outcome the effect measure(s) (e.g. risk ratio, mean difference) used in the synthesis or presentation of results. | | Page  6 |
| Synthesis methods | 13a | Describe the processes used to decide which studies were eligible for each synthesis (e.g. tabulating the study intervention characteristics and comparing against the planned groups for each synthesis (item #5)). | | Pages 5-6 |
|  | 13b | Describe any methods required to prepare the data for presentation or synthesis, such as handling of missing summary statistics, or data conversions. | | Pages 5 |
|  | 13c | Describe any methods used to tabulate or visually display results of individual studies and syntheses. | | Page  5-6 |
|  | 13d | Describe any methods used to synthesize results and provide a rationale for the choice(s). If meta-analysis was performed, describe the model(s), method(s) to identify the presence and extent of statistical heterogeneity, and software package(s) used. | | Page  6 |
|  | 13e | Describe any methods used to explore possible causes of heterogeneity among study results (e.g. subgroup analysis, meta-regression). | | Pages  6 |
|  | 13f | Describe any sensitivity analyses conducted to assess robustness of the synthesized results. | | Pages 6 |
| Reporting bias assessment | 14 | Describe any methods used to assess risk of bias due to missing results in a synthesis (arising from reporting biases). | | Pages 6 |
| Certainty assessment | 15 | Describe any methods used to assess certainty (or confidence) in the body of evidence for an outcome. | | Pages  6 |
| RESULTS | | | |  |
| Study selection | 16a | Describe the results of the search and selection process, from the number of records identified in the search to the number of studies included in the review, ideally using a flow diagram. | | Page  8  Figure 1 |
|  | 16b | Cite studies that might appear to meet the inclusion criteria, but which were excluded, and explain why they were excluded. | Page 8 | |
| Study characteristics | 17 | Cite each included study and present its characteristics. | | Pages 8-9  Table 1 |
| Results of individual studies | 19 | For all outcomes, present, for each study: (a) summary statistics for each group (where appropriate) and (b) an effect estimate and its precision (e.g. confidence/credible interval), ideally using structured tables or plots. | | Pages  9-10  Figure 2 |
| Results of syntheses | 20a | Present results of all statistical syntheses conducted. If meta-analysis was done, present for each the summary estimate and its precision (e.g. confidence/credible interval) and measures of statistical heterogeneity. If comparing groups, describe the direction of the effect. | | Pages 9-10  Figure 2 |
|  | 20b | Present results of all investigations of possible causes of heterogeneity among study results. | | Page 10-12  Figure 3, 4 |
|  | 20c | Present results of all sensitivity analyses conducted to assess the robustness of the synthesized results. | | Page 10-12 |
| Reporting biases | 21 | Present assessments of risk of bias due to missing results (arising from reporting biases) for each synthesis assessed. | | Page 12 |
| DISCUSSION | | | |  |
| Discussion | 23a | Provide a general interpretation of the results in the context of other evidence. | | Pages  13-14 |
|  | 23b | Discuss any limitations of the evidence included in the review. | | Page 14-15 |
|  | 23c | Discuss any limitations of the review processes used. | | Page 14-15 |
|  | 23d | Discuss implications of the results for practice, policy, and future research. | | Page 13-15 |
| OTHER INFORMATION | | | |  |
| Registration and protocol | 24a | Provide registration information for the review, including register name and registration number, or state that the review was not registered. | | Page 5 |
|  | 24b | Indicate where the review protocol can be accessed, or state that a protocol was not prepared. | | Page 5 |
|  | 24c | Describe and explain any amendments to information provided at registration or in the protocol. | | Page 5 |
| Support | 25 | Describe sources of financial or non-financial support for the review, and the role of the funders or sponsors in the review. | | Page 15 |
| Competing interests | 26 | Declare any competing interests of review authors. | | Page 15 |
| Availability of data, code and other materials | 27 | Report which of the following are publicly available and where they can be found: template data collection forms; data extracted from included studies; data used for all analyses; analytic code; any other materials used in the review. | | Page 15 |

**Supplementary Table 2.** PRISMA 2020 for Abstract Checklist.

| Section and Topic | Item # | Checklist item | Reported (Yes/No) |
| --- | --- | --- | --- |
| TITLE | | |  |
| Title | 1 | Identify the report as a systematic review. | Yes |
| BACKGROUND | | |  |
| Objectives | 2 | Provide an explicit statement of the main objective(s) or question(s) the review addresses. | Yes |
| METHODS | | |  |
| Eligibility criteria | 3 | Specify the inclusion and exclusion criteria for the review. | Yes |
| Information sources | 4 | Specify the information sources (e.g. databases, registers) used to identify studies and the date when each was last searched. | Yes |
| Risk of bias | 5 | Specify the methods used to assess risk of bias in the included studies. | Yes |
| Synthesis of results | 6 | Specify the methods used to present and synthesise results. | No |
| RESULTS | | |  |
| Included studies | 7 | Give the total number of included studies and participants and summarise relevant characteristics of studies. | Yes |
| Synthesis of results | 8 | Present results for main outcomes, preferably indicating the number of included studies and participants for each. If meta-analysis was done, report the summary estimate and confidence/credible interval. If comparing groups, indicate the direction of the effect (i.e. which group is favoured). | Yes |
| DISCUSSION | | |  |
| Limitations of evidence | 9 | Provide a brief summary of the limitations of the evidence included in the review (e.g. study risk of bias, inconsistency and imprecision). | No |
| Interpretation | 10 | Provide a general interpretation of the results and important implications. | Yes |
| OTHER | | |  |
| Funding | 11 | Specify the primary source of funding for the review. | No |
| Registration | 12 | Provide the register name and registration number. | Yes |

**Supplementary Table 3.** Inclusion and exclusion criteria of included studies.

| **Study** | **Inclusion Criteria** | **Exclusion Criteria** |
| --- | --- | --- |
| ^Krishnan, 2024^ | 1. All individuals aged 18 years or older 2. Initial mastitis diagnosis (ICD-10: N61) from 315 office-based gynecologists in Germany between January 2005and December 2021 3. Observation time of a least 12 months prior to the index date and no cancer diagnoses (ICD-10: C00-C97) 4. No benign, in situ or uncertain behavior neoplasms of the breast (ICD-10: D05, D24, D48.6, D49.3) prior to or at index date, or within six months after the index date. |  |
| ^Bothou, 2022^ | 1. women with histologically confirmed breast cancer history |  |
| Chen, 2020 | 1. Women newly diagnosed withmastitis mastitis (ICD-9-CM code = 611.0) from 2010 to 2012 2. Women older than 40 years old 3. The non-mastitis patients were defined as not having had a diagnosis of mastitis (ICD-9-CM codes = 611.0, 675) from 2009 to 2013. | 1. Diagnoses of breast cancer (ICD-9-CM code = 174) before the index |
| Chang, 2019 | 1. ICD-9 codes (ICD-9: 611.0 and 675) to identify the mastitis group: 2. Women who were newly diagnosed with mastitis or abscess between 2000 and 2011. 3. Women who had at least two out- patient visits or one hospitalisation due to mastitis or abscess were included. | 1. Non-lactational mastitis cohort:  - Women who had lactational mastitis, women who had any - Pre-existing cancer before the index date, women who had any cancer including breast cancer within 1 year after - The index date, women who did not have basic demographic information, women less than 20 years of age and  2. Pre-existing cancer 3. Mastitis associated with breastfeeding 4. Women with any cancer within one year after the index date. 5. Missing demographic information 6. Age <20 |

**Supplementary Table 4.** Search Strategies.

| **Database** | **Search Strategy** |
| --- | --- |
| **PubMed** | ("Breast Neoplasm" OR "Neoplasm, Breast" OR "Neoplasms, Breast" OR "Breast Tumors" OR "Breast Tumor" OR "Tumor, Breast" OR "Tumors, Breast" OR "Breast Cancer" OR "Cancer, Breast" OR "Cancer of Breast" OR "Cancer of the Breast" OR "Malignant Neoplasm of Breast" OR "Breast Malignant Neoplasm" OR "Breast Malignant Neoplasms" OR "Malignant Tumor of Breast" OF "Breast Malignant Tumor" OR "Breast Malignant Tumors" OR "Mammary Cancer" OR "Cancer, Mammary" OR "Cancers, Mammary" OR "Mammary Cancers" OR "Mammary Neoplasms, Human" OR "Human Mammary Neoplasm" OR "Human Mammary Neoplasms" OR "Neoplasm, Human Mammary" OR "Neoplasms, Human Mammary" OR "Mammary Neoplasm, Human" OR "Breast Carcinoma" OR "Breast Carcinomas" OR "Carcinoma, Breast" OR "Carcinomas, Breast" OR "Mammary Carcinoma, Human" OR "Carcinoma, Human Mammary" OR "Carcinomas, Human Mammary" OR "Human Mammary Carcinomas" OR "Mammary Carcinomas, Human" OR "Human Mammary Carcinoma") AND (mastitis OR Granulomatous mastitis OR lactation mastitis OR non lactating mastitis) AND (risk OR association) AND (women or lachant women or non lactant and women) |
| **Embase** | TITLE-ABS-KEY ("Breast Neoplasm") OR TITLE-ABS-KEY ("Neoplasm, Breast") OR TITLE-ABS-KEY ("Neoplasms, Breast") OR TITLE-ABS-KEY ("Breast Tumors") OR TITLE-ABS-KEY ("Breast Tumor") OR TITLE-ABS-KEY ("Tumor, Breast") OR TITLE-ABS-KEY ("Tumors, Breast") OR TITLE-ABS-KEY ("Breast Cancer") OR TITLE-ABS-KEY ("Cancer, Breast") OR TITLE-ABS-KEY ("Cancer of Breast") OR TITLE-ABS-KEY ("Cancer of the Breast") OR TITLE-ABS-KEY ("Malignant Neoplasm of Breast") OR TITLE-ABS-KEY ("Breast Malignant Neoplasm") OR TITLE-ABS-KEY ("Breast Malignant Neoplasms") OR TITLE-ABS-KEY ("Malignant Tumor of Breast") OF TITLE-ABS-KEY ("Breast Malignant Tumor") OR TITLE-ABS-KEY ("Breast Malignant Tumors") OR TITLE-ABS-KEY ("Mammary Cancer") OR TITLE-ABS-KEY ("Cancer, Mammary") OR TITLE-ABS-KEY ("Cancers, Mammary") OR TITLE-ABS-KEY ("Mammary Cancers") OR TITLE-ABS-KEY ("Mammary Neoplasms, Human") OR TITLE-ABS-KEY ("Human Mammary Neoplasm") OR TITLE-ABS-KEY ("Human Mammary Neoplasms") OR TITLE-ABS-KEY ("Neoplasm, Human Mammary") OR TITLE-ABS-KEY ("Neoplasms, Human Mammary") OR TITLE-ABS-KEY ("Mammary Neoplasm, Human") OR TITLE-ABS-KEY ("Breast Carcinoma") OR TITLE-ABS-KEY ("Breast Carcinomas") OR TITLE-ABS-KEY ("Carcinoma, Breast") OR TITLE-ABS-KEY ("Carcinomas, Breast") OR TITLE-ABS-KEY ("Mammary Carcinoma, Human") OR TITLE-ABS-KEY ("Carcinoma, Human Mammary") OR TITLE-ABS-KEY ("Carcinomas, Human Mammary") OR TITLE-ABS-KEY ("Human Mammary Carcinomas") OR TITLE-ABS-KEY ("Mammary Carcinomas, Human") OR TITLE-ABS-KEY ("Human Mammary Carcinoma")) AND TITLE-ABS-KEY (mastitis) OR TITLE-ABS-KEY (Granulomatous mastitis) OR TITLE-ABS-KEY (lactation mastitis) OR TITLE-ABS-KEY (non lactating mastitis)) AND TITLE-ABS-KEY (risk) OR TITLE-ABS-KEY (association)) AND (women) OR TITLE-ABS-KEY (women or lachant women or non lactant and women) OR TITLE-ABS-KEY lachant women or non lactant and women)) |
| **The Cochrane Library** | (("Breast Neoplasm"):ti,ab,kw OR ("Neoplasm, Breast"):ti,ab,kw OR ("Neoplasms, Breast"):ti,ab,kw OR ("Breast Tumors"):ti,ab,kw OR ("Breast Tumor"):ti,ab,kw OR ("Tumor, Breast"):ti,ab,kw OR ("Tumors, Breast"):ti,ab,kw OR ("Breast Cancer"):ti,ab,kw OR ("Cancer, Breast"):ti,ab,kw OR ("Cancer of Breast"):ti,ab,kw OR ("Cancer of the Breast"):ti,ab,kw OR ("Malignant Neoplasm of Breast"):ti,ab,kw OR ("Breast Malignant Neoplasm"):ti,ab,kw OR ("Breast Malignant Neoplasms"):ti,ab,kw OR ("Malignant Tumor of Breast"):ti,ab,kw OR ("Breast Malignant Tumor"):ti,ab,kw OR ("Breast Malignant Tumors"):ti,ab,kw OR ("Mammary Cancer"):ti,ab,kw OR ("Cancer, Mammary"):ti,ab,kw OR ("Cancers, Mammary"):ti,ab,kw OR ("Mammary Cancers"):ti,ab,kw OR ("Mammary Neoplasms, Human"):ti,ab,kw OR ("Human Mammary Neoplasm"):ti,ab,kw OR ("Human Mammary Neoplasms"):ti,ab,kw OR ("Neoplasm, Human Mammary"):ti,ab,kw OR ("Neoplasms, Human Mammary"):ti,ab,kw OR ("Mammary Neoplasm, Human"):ti,ab,kw OR ("Breast Carcinoma"):ti,ab,kw OR ("Breast Carcinomas"):ti,ab,kw OR ("Carcinoma, Breast"):ti,ab,kw OR ("Carcinomas, Breast"):ti,ab,kw OR ("Mammary Carcinoma, Human"):ti,ab,kw OR ("Carcinoma, Human Mammary"):ti,ab,kw OR ("Carcinomas, Human Mammary"):ti,ab,kw OR ("Human Mammary Carcinomas"):ti,ab,kw OR ("Mammary Carcinomas, Human"):ti,ab,kw OR ("Human Mammary Carcinoma"):ti,ab,kw) AND ((mastitis OR Granulomatous mastitis):ti,ab,kw OR (lactation mastitis):ti,ab,kw OR (non lactating mastitis):ti,ab,kw) AND ((risk):ti,ab,kw OR (association):ti,ab,kw) AND ((women):ti,ab,kw OR (lachant women):ti,ab,kw OR (non lactant and women):ti,ab,kw) |
